# Linking single nucleotide polymorphisms to signaling blueprints in abdominal aortic aneurysms

**DOI:** 10.1038/s41598-022-25144-y

**Published:** 2022-12-05

**Authors:** Chrysania Lim, Muhammad Yogi Pratama, Cristobal Rivera, Michele Silvestro, Philip S. Tsao, Lars Maegdefessel, Katherine A. Gallagher, Thomas Maldonado, Bhama Ramkhelawon

**Affiliations:** 1 Division of Vascular and Endovascular Surgery, Department of Surgery, New York University Langone Medical Center, New York, USA; 2 Department of Biomedicine, Indonesia International Institute for Life-Sciences (i3L), Jakarta, Indonesia; 3 Department of Cell Biology, New York University Langone Medical Center, New York, USA; 4 VA Palo Alto Health Care System, Palo Alto, CA USA; 5 Department of Medicine, Stanford University School of Medicine, Stanford, CA USA; 6 Department of Vascular and Endovascular Surgery, Technical University Munich, Munich, Germany; 7 German Center for Cardiovascular Research (DZHK), Partner Site Munich Heart Alliance, Berlin, Germany; 8 Department of Medicine, Karolinska Institute, Stockholm, Sweden; 9 Department of Surgery, University of Michigan, Ann Arbor, MI USA

**Keywords:** Genome-wide association studies, Aneurysm, Cardiovascular genetics, Gene regulatory networks, Cellular signalling networks

## Abstract

Abdominal aortic aneurysms (AAA) is a multifactorial complex disease with life-threatening consequences. While Genome-wide association studies (GWAS) have revealed several single nucleotide polymorphisms (SNPs) located in the genome of individuals with AAA, the link between SNPs with the associated pathological signals, the influence of risk factors on their distribution and their combined analysis is not fully understood. We integrated 86 AAA SNPs from GWAS and clinical cohorts from the literature to determine their phenotypical vulnerabilities and association with AAA risk factors. The SNPs were annotated using snpXplorer AnnotateMe tool to identify their chromosomal position, minor allele frequency, CADD (Combined Annotation Dependent Depletion), annotation-based pathogenicity score, variant consequence, and their associated gene. Gene enrichment analysis was performed using Gene Ontology and clustered using REVIGO. The plug-in GeneMANIA in Cytoscape was applied to identify network integration with associated genes and functions. 15 SNPs affecting 20 genes with a CADD score above ten were identified. AAA SNPs were predominantly located on chromosome 3 and 9. Stop-gained rs5516 SNP obtained high frequency in AAA and associated with proinflammatory and vascular remodeling phenotypes. SNPs presence positively correlated with hypertension, dyslipidemia and smoking history. GO showed that AAA SNPs and their associated genes could regulate lipid metabolism, extracellular matrix organization, smooth muscle cell proliferation, and oxidative stress, suggesting that part of these AAA traits could stem from genetic abnormalities. We show a library of inborn SNPs and associated genes that manifest in AAA. We uncover their pathological signaling trajectories that likely fuel AAA development.

## Introduction

Abdominal aortic aneurysms (AAA) are characterized by localized weakening and dilation of the abdominal aorta^1^ that results from a cascade of mechanisms including transmural inflammation^2^, smooth muscle cell (SMC) apoptosis^3^ and extracellular matrix (ECM) degradation^4^ that collectively lead to the loss of aortic wall elasticity. AAA often coexists with other cardiovascular diseases^5–7^ and risk factors such as aging and smoking^8^. However, the physiopathology of AAA is uniquely distinguished by signaling programs that result in the exaggerated degradation of the core constituents of the elements of the ECM that lead to life-threatening aortic rupture.

SNPs are single base-pair variations that occur in the DNA either within or outside the coding region of genes and have the potential to interfere at different steps of gene expression depending on their genomic location^9^. For example, SNPs present in non-coding segments of the genome have been shown to modulate the efficacy of gene transcription by impeding the accessibility of transcription factors in these response elements^10^. The presence of SNPs in coding region of genes can give rise to mRNA with different bases at SNP site which could impact the proper translation of mRNA while the presence of SNPs within a coding sequence can lead to an amino-acid change and protein-misfolding^9,11^. Recently, it has been shown that the identification and estimation of variance by all SNPs from GWAS of conventionally unrelated individuals might be a significant determinant of genetic heritability to particular diseases^12,13^. Notably, previous genetic studies have reported that AAA occurrence can be heritable within families, reaching up to a 20% increase in AAA susceptibility within first-degree relatives^14–16^. Notably, a population-based twin study estimated the heritability of AAA to be as strong as between 70 and 77%^17^. However, while the inheritance of SNPs in AAA has not been directly studied in family cohorts, several gene polymorphisms such as *COL3A1*, *MYH11*, and *TGFBR2* were also identified to be transmitted within a familial lineage of AAA^18^. Despite this evidence pointing to the familial risk associated with AAA, there is a paucity of information linking these genetic signatures and their underlying mechanistic significance in the development of AAA.

GWAS have facilitated the accessibility of disease-specific SNPs^19^. Identification of SNPs as predictive markers for disease risk has been used for Alzheimer’s^20^, migraines^21^, and coronary artery disease^22^. In AAA, GWAS of the Million Veteran Program has examined the genetic associations of genetic variants in a subset of AAA independent of family history^23^. As such, identification of AAA-specific SNPs would be beneficial to further characterize the pathogenic pathway associated with each SNP and refine our understanding of the knowledge gap between genomic inheritance and the molecular trajectory that lead to the degradation of the aortic wall that occurs during AAA.

In the current study, we performed an integrative analysis combining the available GWAS catalogs in AAA along with non-GWAS gene association studies published in the literature. We identified 86 SNPs related to AAA and its associated risk factors. We curated this result using multiple bioinformatic analyses to expose their potential phenotypical signals via which they could contribute to AAA development.

## Methods

### Literature search

**Figure 1:**
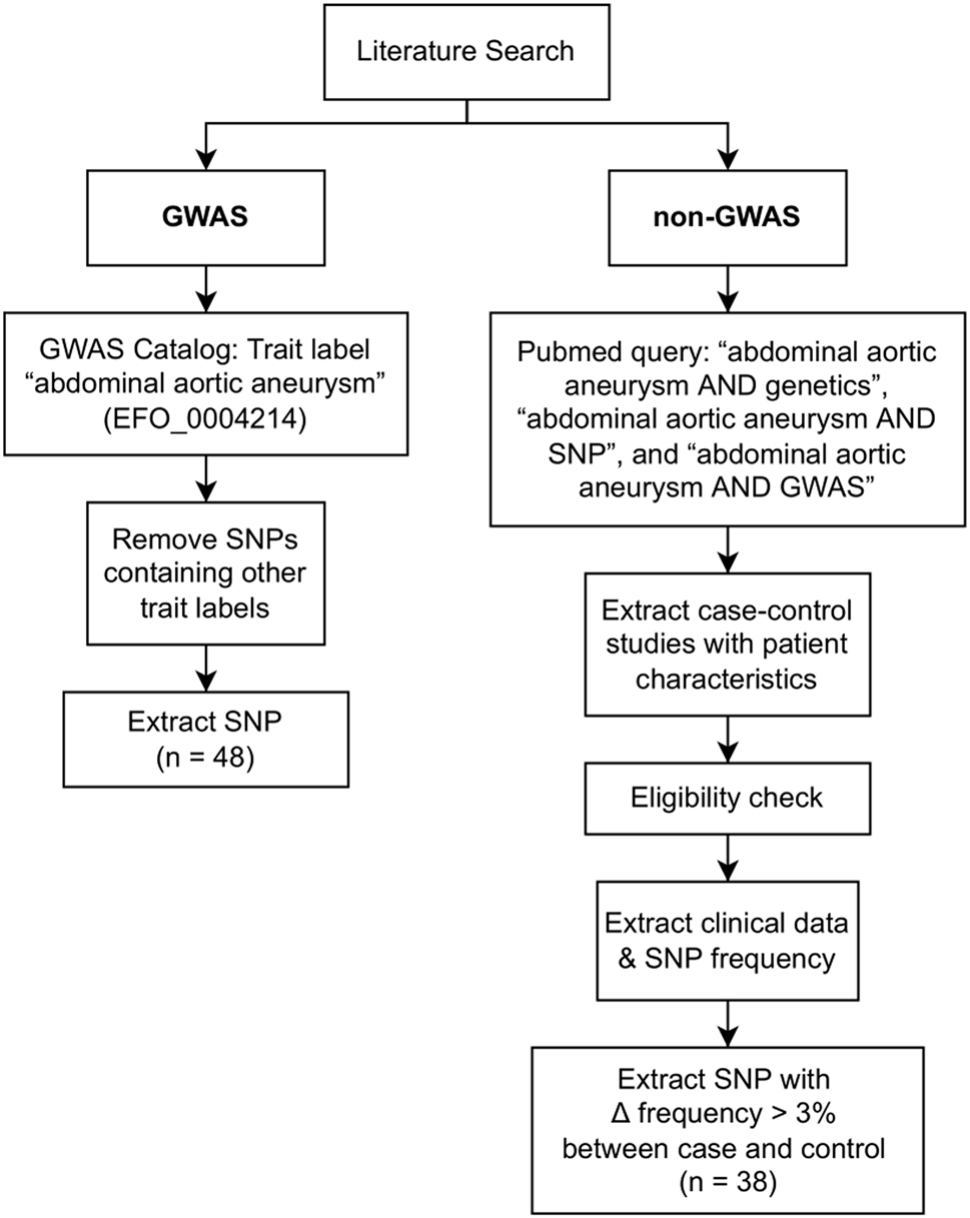
Flow diagram of study inclusion for the analysis.

Identification of studies was conducted through a literature search of PubMed, GWAS central, and GWAS registry. The search query used to retrieve potentially eligible studies from PubMed was “abdominal aortic aneurysm AND genetics”, “abdominal aortic aneurysm AND SNPs”, and “abdominal aortic aneurysm AND GWAS”. In addition, we searched for other possible SNPs outside of GWAS central and GWAS catalog by considering AAA candidate gene association studies with detailed clinical characteristics, reporting data on the associations between AAA and different SNPs. We only included published full-text articles until April 2022. The flow diagram of the study inclusion is described in Fig. 1.

### Selection criteria and quality assessment

GWAS studies were included if they were listed in the GWAS directory with the trait label “abdominal aortic aneurysm” (EFO_0004214), with no other labels. Non-GWAS studies were considered eligible if they evaluated the association between AAA and genetic polymorphism through effect measures of odds ratio (OR), with 95% confidence interval (CI); if they reported allele and minor allele frequency (MAF) of population groups; and if controls were in Hardy–Weinberg equilibrium. We excluded the studies with data published only in abstract form, studies where MAF in controls were lower than 1% (rare variants), with sample size fewer than 10 cases or controls, studies that were rebutted by others and studies that combined data with other cardiovascular diseases. systematic reviews and animal in vivo studies were also not included in our analysis. We based our selection criteria on the principles proposed by the Human Genome Epidemiology Network (HuGeNet) for the meta-analysis of molecular association studies^20^.

The following clinical data were extracted from eligible AAA candidate gene association studies when available: age, gender percentage, aortic diameter (mm), smoking history (past or current), hypertension, diabetes, coronary artery disease (CAD), peripheral artery disease (PAD), and dyslipidemia (or hypercholesterolemia). SNPs frequency and genotype data of case and control groups were also extracted.

### SNPs-gene annotation and pathway enrichment analysis

The SNPs used in Gene-set Enrichment Analysis were obtained from GWAS database with the trait label “abdominal aortic aneurysm” (EFO_0004214) and from other studies where allele frequency in AAA was reported to be 3% more than in controls. The SNPs were annotated using snpXplorer AnnotateMe tool^24^ with the following settings: SNPs-gene annotation or Gene-set Enrichment Analysis, GRCh38, GTEx tissue (Blood, Aorta, Coronary) and GO:BP. For each SNP input, SNPs-gene annotation provided the following information: chromosomal position; MAF; CADD-annotation based pathogenicity score (CADD v1.6), variant consequence, affected gene; GTEx based eQTL (expression quantitative-trait-loci) and sQTL (splicing quantitative-trait-loci); closest affected gene. Input SNPs approximate affected genes based on position within the genome, expression in GTEx tissue, or their direct coding gene.

Gene enrichment analysis was performed on AAA-related genes using Gene Ontology (GO) terms as the gene-set source. Clustering of the enriched GO terms was performed using REVIGO^25^, and annotated terms were selected through a semantic similarity matrix and a dynamic cut tree algorithm for term-based clustering^24^. The plug-in GeneMANIA in Cytoscape was used as an analytical method to provide an association network integration to predict gene function and gene–gene interaction of the SNPs-associated gene in this study^26,27^. GeneMANIA weighted each functional genomic dataset according to its predictive value according to its gene query while suggesting more genes with similar domain structure or physical interaction^27^. Gene interaction was plotted via Cytoscape.

A Mann–Whitney U test was performed using R software^28^ on patient clinical data. Comparisons of age, aortic diameter, male percentage, smoking history (past and current), hypertension, dyslipidemia, diabetes, CAD, and PAD were performed if data was available. Data were plotted using R studio (v.1.3.1093).

## Results

### Distribution of AAA SNPs in the genome and their gene regulation

**Figure 2:**
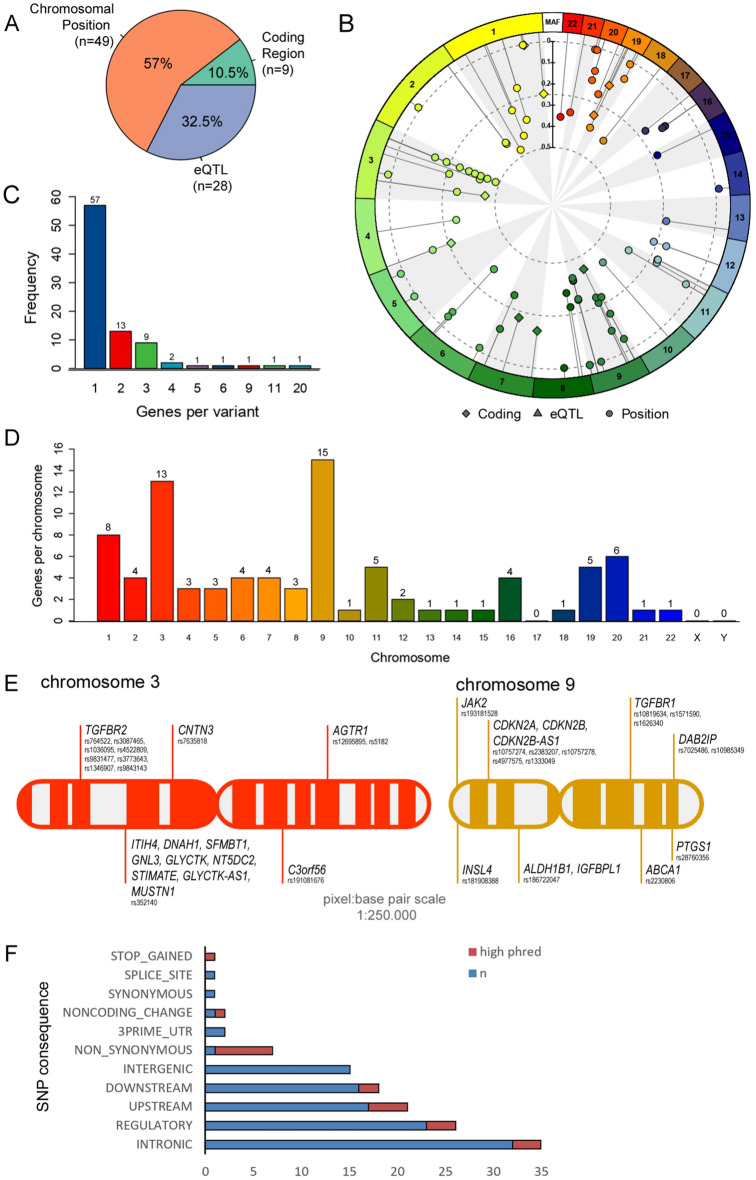
Genome Distribution of 86 SNPs Associated with AAA. (**A**) Most of the identified SNPs were annotated based on their chromosomal position (n = 49), followed by GTEx tissue expression (n = 28) and direct gene coding (n = 9). (**B**) The frequency of genes per genetic variant identified in this study. (**C**) The circular plot analyzed using snpXplorer AnnotateMe tool visualized the annotation type of each genetic variant (coding region, eQTL, or their positions) and their respective minor allele frequency and chromosomal distribution. (**D**) Distribution of SNPs across different chromosomes, with chromosome 3 and 9 having more AAA-related SNPs. (**E**) The complete SNP chromosome mapping of chromosome 3 and 9 as the top two locations of SNPs in AAA using a scale of 1:250,000 scale of pixels. (**F**) SNPs associated with AAA most commonly reside in their respective gene's intronic, regulatory, upstream, downstream, or intergenic regions.

The literature search identified 86 SNPs related to AAA, 48 of which originated from the recorded GWAS databases, and 38 were from AAA gene association studies in which SNPs frequency in AAA patients was at least 3% higher than in the control group. The variant-gene mapping procedure performed with SNPs snpXplorer AnnotateMe tool showed that most of the identified SNPs were annotated based on their chromosomal position (n = 49), followed by their eQTL GTEx tissue expression (n = 28) and direct gene coding region (n = 9) (Fig. 2A,B), implying the possible effect of the SNPs of interest in AAA. The specificity of each SNPs was observed since the majority of the SNPs (*N* = 57 variants) are mapped and annotated for one gene (Fig. 2C). We observed a random distribution of SNPs across different chromosomes. Chromosome 3 and 9 predominantly harbored most of the AAA SNPs (Fig. 2D). No SNPs were found on chromosome 17, X, and Y, suggesting that AAA SNPs identified in this study are not sex-linked (Fig. 2D).

On average, each chromosome contains 4 SNPs, with chromosomes 3 and 9 in contain the most SNPs (Fig. 2E). In the chromosome 3, we found 8 SNPs that affect a single gene (*TGFBR2)* and 1 SNP that affects 9 different genes (*ITIH4, DNAH1, SFMBT1, GNL3, GLYCTK, NT5DC2, STIMATE, GLYCTK-AS1, MUSTN1*). In the chromosome 9, we found 3 SNPs that affect a single gene (TGFBR1). The complete SNP chromosome mapping in a 1:250,000 scale of pixel to base pair can be seen in Supplementary Fig. 1.

Single SNP can affect multiple genes depending on their genomic location. Most of the SNPs associated with AAA reside in the intronic, regulatory, upstream, downstream, or intergenic region of their respective gene (Fig. 2F). In particular, SNPs with high CADD pathogenicity scores (> 10) result in non-synonymous, noncoding change, and stop gained mutations. A high CADD pathogenicity score is indicative of the deleterious effect of the SNP, as compared to other possible mutations within the human genome^29^.

### SNPs and the associated genes are linked with high pathogenicity in AAA

We identified 15 SNPs affecting 20 genes with a CADD (Combined Annotation Dependent Depletion) pathogenicity score above 10 (Table 1). CADD scores correlate with pathogenicity, disease severity, regulatory effects, and complex trait associations. A score greater than 10 indicates that the nucleotide substitution is predicted to be the 10% most deleterious substitution within the human genome, a score of 20 or greater indicates the 1% most deleterious, a score of 30 or greater indicates the 0.1% most deleterious and so on. We found 1 SNP with a CADD score above 30 (rs5516, *KLK1*), 1 SNP with a CADD score above 20 (rs1801133, *MTHFR*), and 13 SNPs with a CADD score above 10.

**Table 1:** The list of SNPs with high CADD (combined annotation dependent depletion) pathogenicity score.

No	SNP ID	Affected Gene(s)	SNP consequence	MAF	CADD pathogenicity score*	Protein-coding SNP (yes/no)
1	rs5516	*KLK1*	STOP_GAINED	0.3073	34	Yes
2	rs1801133	*MTHFR*	NON_SYNONYMOUS	0.2454	25.6	Yes
3	rs429358	*APOE*	NON_SYNONYMOUS	0.1506	17.4	Yes
4	rs2276109	*MMP12*	UPSTREAM	0.05551	17.25	No
5	rs11591147	*PCSK9*	NON_SYNONYMOUS	0.00639	17.03	Yes
6	rs1800795	*STEAP1B, IL6-AS1, IL6*	REGULATORY, INTRONIC	0.1412	16.22	No
7	rs7255	*LDAH, GDF7, C2orf43*	UPSTREAM, NONCODING_CHANGE, DOWNSTREAM	0.4139	14.92	No
8	rs2836411	*ERG*	REGULATORY, INTRONIC	0.3269	14.67	No
9	rs2230806	*ABCA1*	NON_SYNONYMOUS	0.4397	14.3	Yes
10	rs2071307	*ELN*	NON_SYNONYMOUS	0.2204	13.86	Yes
11	rs243865	*MMP2-AS1, MMP2*	UPSTREAM	0.1366	11.61	No
12	rs2285053	*MMP2*	UPSTREAM	0.1512	10.81	No
13	rs1571590	*TGFBR1*	INTRONIC	0.1216	10.78	No
14	rs1799983	*NOS3*	NON_SYNONYMOUS	0.1763	10.55	Yes
15	rs10757278	*CDKN2B, CDKN2B-AS1*	REGULATORY, DOWNSTREAM	0.4081	10.42	No

Higher CADD scores correlate to the deleterious effect of the nucleotide substitution, compared to all possible substitutions within the human genome.

KLK1, Kallikrein 1; MTHFR, Methylenetetrahydrofolate Reductase; APOE, Apolipoprotein E; MMP12, Matrix Metallopeptidase 12; PCSK9, Proprotein Convertase Subtilisin/Kexin Type 9; STEAP1B, STEAP Family Member 1B; IL6-AS1, IL6 Antisense RNA 1; IL6, Interleukin 6; LDAH, Lipid Droplet Associated Hydrolase; GDF7, Growth Differentiation Factor 7; ERG, ETS Transcription Factor ERG; ABCA1, ATP Binding Cassette Subfamily A Member 1; ELN, Elastin; MMP2-AS1, MMP2 Antisense RNA 1; MMP2, Matrix Metallopeptidase 2; TGFBR1, Transforming Growth Factor Beta Receptor 1; NOS3, Nitric Oxide Synthase 3; CDKN2B, Cyclin Dependent Kinase Inhibitor 2B; CDKN2B-AS1, CDKN2B Antisense RNA 1.

*A significant CADD pathogenicity score is defined as a score greater than 10.

We confirmed the association between SNPs with high CADD pathogenicity scores with their frequency on the retrieved 38 AAA-gene association studies (Table 2). We identified four SNPs and associated genes consisting of the SNP rs5516 (*KLK1*), rs1800795 (*IL-6*), rs2230806 (*ABCA1*), and rs243865 (*MMP-2*) have both higher CADD scores and frequency in AAA patients. However, we observed that a high CADD score does not necessarily correlate with frequency in AAA.

**Table 2:** Top 20 upregulated SNPs from the retrieved AAA gene association clinical studies.

No	SNP ID	Affected Gene(s)	Allele/Genotype	AAA frequency (Δ frequency)	CADD score	References
1	rs5516	*KLK1*	CG	57.9 (17.8)	34	Biros et al.^30^
2	rs1800795	*IL-6*	C	51.9 (10.8)	16.22	Jabłońska et al.^31^
3	rs2230806	*ABCA1*	KK	43.6 (13.3)	14.3	Zhao et al.^32^
4	rs243865	*MMP-2*	CC	64.9 (8.6)	11.61	Saracini et al.^33^
5	rs1800629	*TNF-α*	GA	45.2 (16.6)	4.365	Jabłońska et al.^31^
6	rs2071307	*ELN*	GG	39.9 (8.6)	13.86	Saracini et al.^33^
7	rs3091244	*CRP*	CT, CA	47 (14)	0.004	Saratzis et al.^34^
8	rs1800469	*TGFB1*	TT	31.2 (11.5)	5.903	Zuo et al.^35^
9	rs7635818	*CNTN3*	CC	27.1 (11.2)	1.023	Rašiová et al.^36^
10	rs3091244	*CRP*	TT, AA, TA	20 (11)	0.004	Saratzis et al.^37^
11	rs3775290	*TLR3*	C	68.3 (10.7)	9.846	Jabłońska et al.^38^
12	rs1333049	*CDKN2B, CDKN2B-AS1*	CC	31.6 (10.6)	1.579	Wei et al.^39^
13	rs10757278	*CDKN2B, CDKN2B-AS1*	G	56.5 (8.8)	10.42	Wei et al.^39^
14	rs5516	*KLK1*	GG	15 (9)	34	Biros et al.^30^
15	rs352140	*TLR9*	T	52.4 (8.6)	0.066	Jabłońska et al.^38^
16	rs1466535	*LRP1*	CT	43 (8)	6.903	Galora et al.^40^
17	rs3918242	*MMP-9*	CT	29.9 (7.7)	0.056	Crkvenac Gregorek et al.^41^
18	rs3019885	*SLC30A8*	TT	39.7 (7.5)	6.938	Galora et al.^40^
19	rs2252070	*MMP-13*	GG	21.4 (7.3)	9.775	Saracini et al.^33^
20	rs5182	*AGTR1*	CT	46.5 (6.5)	0.64	Zuo et al.^42^

SNPs with both High CADD score and frequency are highlighted green in the table, implying their association with pathogenicity, disease severity, regulatory effects, and complex trait in AAA.

KLK1, Kallikrein 1; TNF-α, Tumor Necrosis Factor Alpha; CRP, C-reactive Protein; ABCA1, ATP Binding Cassette Subfamily A Member 1; TGFB1, Transforming Growth Factor Beta-1; CNTN3, Contactin-3; IL6, Interleukin 6; TLR3, Toll-like receptor 3; CDKN2B, Cyclin Dependent Kinase Inhibitor 2B; CDKN2B-AS1, CDKN2B Antisense RNA 1; MMP2, Matrix Metallopeptidase 2; TLR9, Toll-like receptor 9; ELN, Elastin; LRP1, Low Density Lipoprotein Receptor-related Protein 1; MMP9, Matrix Metallopeptidase 9; SLC30A8, Solute Carrier Family 30 Member 8; MMP13, Matrix Metallopeptidase 13; AGTR1, Angiotensin II Receptor Type 1.

### Biological traits associated with AAA SNPs

**Figure 3:**
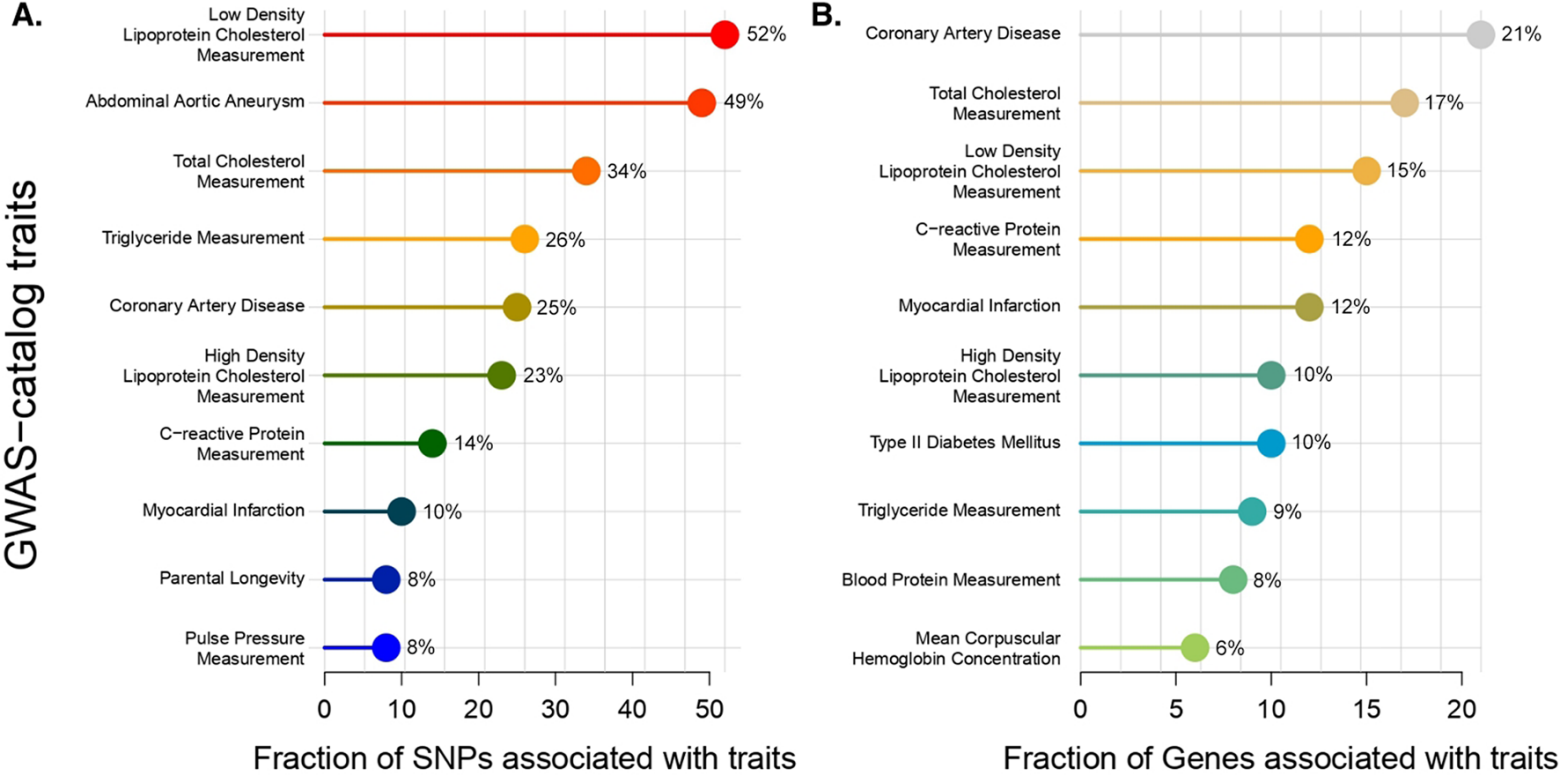
GWAS traits of AAA SNPs. GWAS-catalog Traits of 86 SNPs and Genes Associated with AAA, shown in a fraction of (**A**) total SNP (86) or (**B**) genes (130). The numbers presented are fractions of the GWAS-catalog traits from all tested SNPs or genes.

Using SnpXplorer AnnotateMe platform, gene enrichment analysis from the GWAS-catalog associations of the AAA SNPs showed strong correlations with various lipid metabolism pathways such as LDL-cholesterol measurement (52%), total cholesterol measurement (34%), triglyceride measurement (26%), HDL-cholesterol measurement (23%), and CRP measurement (14%) (Fig. 3A). Only 49% of all tested SNPs were found to be directly labeled with the AAA trait, this may suggest that the remaining 51% have an indirect association with AAA occurrence through various lipid metabolism pathways and genes. As for associations with other cardiovascular diseases, AAA and CAD share 25% of the same SNPs, while AAA and MI only share 10%.

In addition to SNPs, we present the GWAS-catalog associations of the genes associated with AAA SNPs. AAA-SNPs associated genes have correlations with various diseases such as coronary artery disease (21%), myocardial infarction (12%), and type II diabetes (10%). AAA SNPs-associated genes were also found to be associated with genes relating to total cholesterol measurement (17%), LDL-cholesterol measurement (15%), CRP measurement (12%), HDL-cholesterol measurement (10%), and triglyceride measurement (9%) (Fig. 3B).

### Gene ontology analysis of AAA SNPs

**Figure 4:**
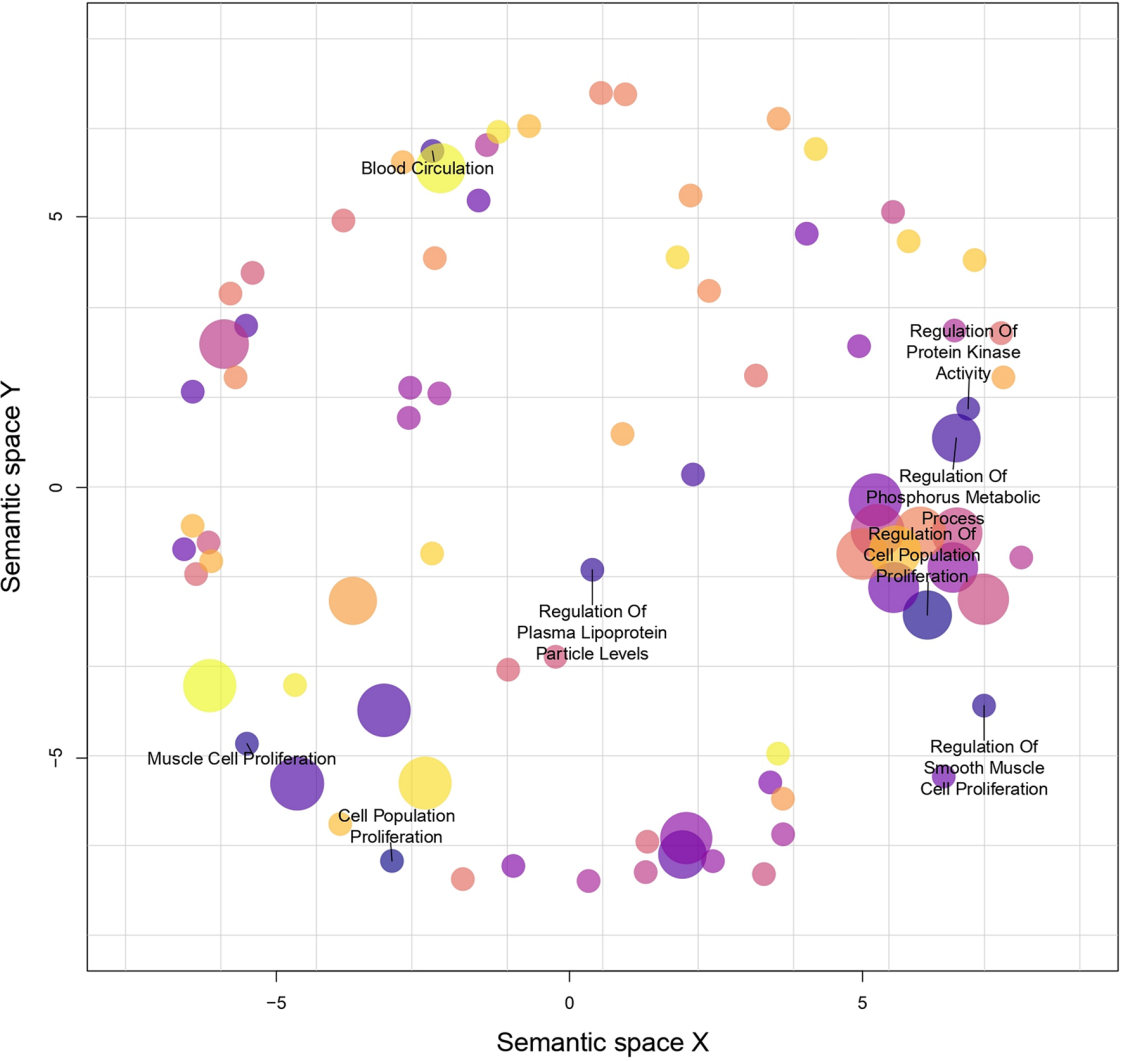
REVIGO Gene Ontology term clustering of 130 AAA genes. The position of each cluster within the semantic space does not matter. Semantically similar GO terms are positioned closer together in the plot.

Gene-set enrichment analysis was done with the genes associated with AAA SNPs, using Gene Ontology (GO) as the gene-set source. The gene-set enrichment was then visualized using REVIGO^25^. Annotated GO terms were selected using a 2-step process as described by Tesi et al. (2021) in the snpXplorer web server^24^. The GO term clustering and annotation in Fig. 4 depict the most prominent and significant biological processes and associated genes in AAA based on the snpXplorer annotation algorithm.

The AAA-associated genes were found to be prominently involved in cell population proliferation, followed by regulation of cell population proliferation (*p* = 3.19 × 10^−8^), muscle cell proliferation (*p* = 3.08 × 10^−6^), regulation of smooth muscle cell proliferation (*p* = 4.97 × 10^−6^), regulation of plasma lipoprotein particle levels (*p* = 1.58 × 10^−5^), regulation of protein kinase activity (*p* = 2.15 × 10^−5^), regulation of phosphorus metabolic process (*p* = 2.77 × 10^−5^), and blood circulation (*p* = 4.63 × 10^−5^). Images processed using SNPsXplorer showing that these annotated terms were found to be most significant according to the semantic similarity (Supplementary Fig. 2) and a dynamic cut tree algorithm for term-based clustering and p values (Supplementary Fig. 3). The most significant gene ontology terms associated with the SNPs associated genes are summarized in Table 3.

**Table 3:** List of top 20 gene ontology terms associated with the total SNPs associated genes in AAA.

Term ID	Term description	*P* value
GO:0008283	**Cell population proliferation**	2.33 × 10^−8^
GO:0042127	**Regulation of cell population proliferation**	3.19 × 10^−8^
GO:0033002	**Muscle cell proliferation**	3.08 × 10^−6^
GO:0048660	**Regulation of smooth muscle cell proliferation**	4.97 × 10^−6^
GO:0048659	Smooth muscle cell proliferation	5.06 × 10^−6^
GO:0008285	Negative regulation of cell population proliferation	1.37 × 10^−5^
GO:0097006	**Regulation of plasma lipoprotein particle levels**	1.58 × 10^−5^
GO:0045859	**Regulation of protein kinase activity**	2.15 × 10^−5^
GO:0019220	Regulation of phosphate metabolic process	2.77 × 10^−5^
GO:0051174	**Regulation of phosphorus metabolic process**	2.77 × 10^−5^
GO:1901700	Response to oxygen-containing compound	3.06 × 10^−5^
GO:0001932	Regulation of protein phosphorylation	3.73 × 10^−5^
GO:0010033	Response to organic substance	3.83 × 10^−5^
GO:0008015	**Blood circulation**	4.63 × 10^–5^
GO:0050673	Epithelial cell proliferation	5.39 × 10^−5^
GO:0043549	Regulation of kinase activity	6.82 × 10^−5^
GO:0032270	Positive regulation of cellular protein metabolic process	7.19 × 10^−5^
GO:1901698	Response to nitrogen compound	7.37 × 10^−5^
GO:1905952	Regulation of lipid localization	8.05 × 10^−5^
GO:0003013	Circulatory system process	8.80 × 10^−5^

Bolded terms are annotated in the REVIGO clustering from Fig. 4.

*P value was measured using the gost function in gprofiler2 R package (cumulative hypergeometric p-value).

### Significant signaling networks associated with AAA SNPs

**Figure 5:**
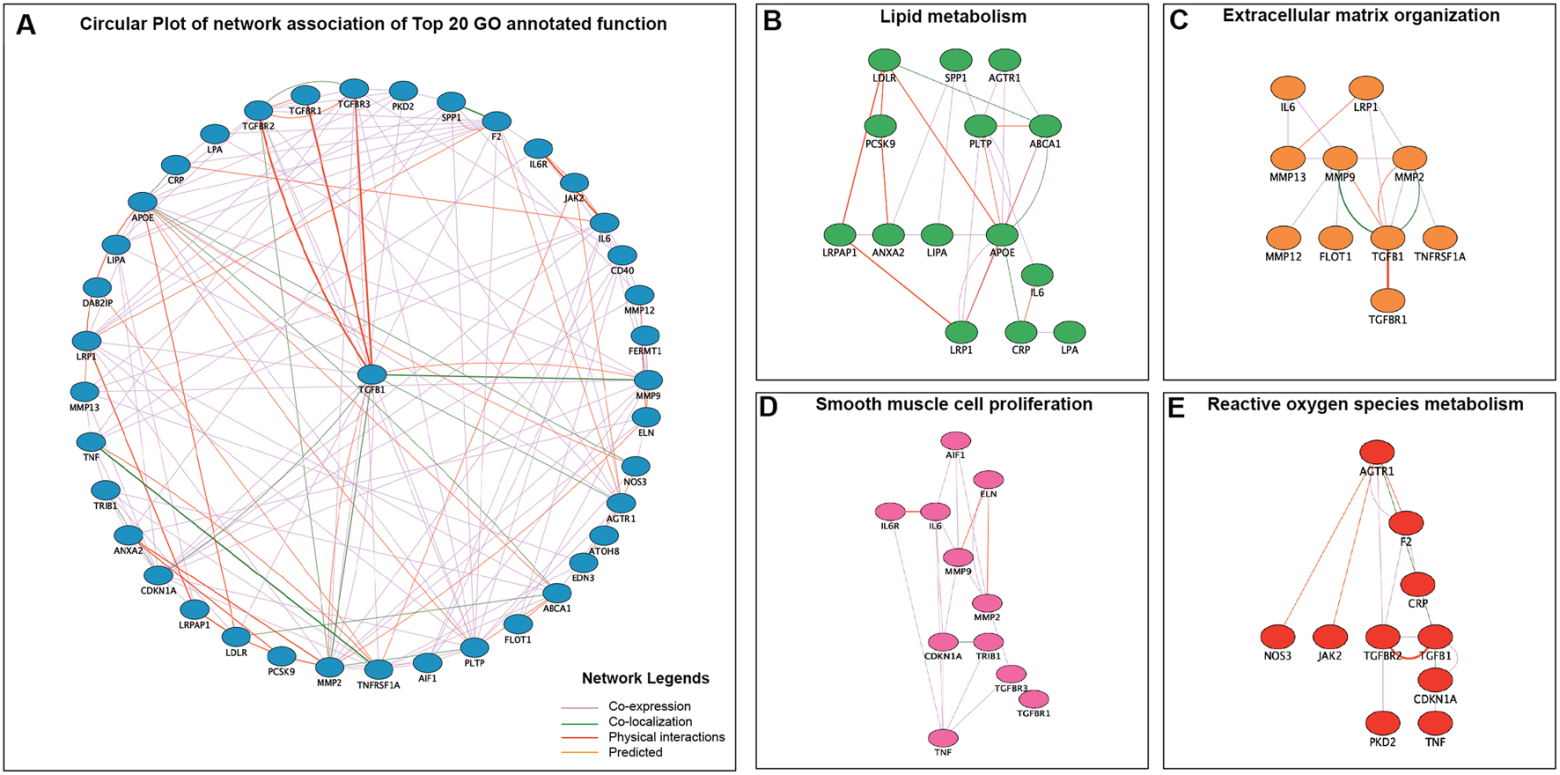
GeneMANIA interaction network from the SNPs associated genes in AAA. (**A**) Circular plot of network association according to top 20 gene ontology annotated function. Transforming growth factor-beta (TGFB1) shares strong physical interaction with its receptor; transforming growth factor beta receptor 1, 2, and 3 (*TGFBR1*, *TGFBR2*, *TGFBR3*) as well as a strong co-localization with matrix metalloproteinase 9 (*MMP9*). The network of hub genes associated with AAA-related pathways consists of (**B**) lipid metabolism pathway, (**C**) extracellular matrix (ECM) organization, (**D**) smooth muscle cell proliferation, and (**E**) reactive oxygen species (ROS) metabolism is hierarchically plotted to show their interaction. The color depth of nodes represents the corrected P-value.

To assess the interaction between SNPs associated genes related to AAA, gene–gene interactomes were constructed and plotted using GeneMANIA plugins in Cytoscape (Fig. 5). The most significant Genes related to top 25 GO annotated functions (Table 4) were included to further grouped and visualized on the network module to see their network association according to their shared pathways, co-localization, co-expression, and physical interaction (Fig. 5A). As a hallmark of pathological remodeling in AAA, we observed the strongest interaction between *TGFB1* with the *TGFB1* receptor family and notably strong co-localization with *MMP2* and *MMP9*, suggesting the role of *TGFB1* pathway as one of the reminiscent factors that could link the presence of SNPs to the development of AAA. This analysis also includes several additional genes predicted to convey a strong interaction with some of our gene candidates. 14 genes were associated with lipid metabolism pathways; regulation of plasma lipoprotein level, regulation of lipid localization, regulation of lipid transport, and regulation of cholesterol transport (Fig. 5B). 10 genes were associated with extracellular matrix (ECM) organization (Fig. 5C). 11 genes were associated with smooth muscle cell proliferation pathways (Fig. 5D). 10 genes were associated with reactive oxygen species metabolism (Fig. 5E). IL-6, TGFB1, and TGFB1 receptor family shared three modules, the most out of all genes. IL-6 is involved in lipid metabolism, ECM organization, and smooth muscle cell proliferation pathways. While TGFB1 and the TGFB1 receptor family are involved in ECM organization, smooth muscle cell proliferation, and ROS metabolism pathways.

**Table 4:** The enriched Gene ontology (GO) term and pathways from the SNPs associated gene queries according to the GENEmania plugin of Cytoscape.

Pathways	ID	Term	Genes	*P*-value
**Lipid metabolism**	GO: 0097006	Regulation of plasma lipoprotein level	*PCSK9, LIPA, PLTP, LRPAP1, LPA, LDLR*	2.1E-06
	GO: 1905952	Regulation of lipid localization	*SPP1, IL6, PCSK9, PLTP, ANXA2, AGTR1, CRP, LDLR, LRP1,ABCA1, APOE*	4.5E-06
	GO: 0032368	Regulation of lipid transport	*PCSK9, LIPA, PLTP, LRPAP1, LPA, LDLR*	0.00036
	GO: 0032374	Regulation of cholesterol transport	*PCSK9, PLTP, ANXA2, LRP1, ABCA1, APOE*	0.0010
**Extracellular matrix organization**	GO: 0030198	Extracellular matrix organization	*IL6, LRP1, MMP13, MMP9, MMP2, MMP12, FLOT1, TGFB1, TGFBR1, TNFRSF1A*	0.00036
**Smooth muscle cell proliferation**	GO: 0033002	Muscle cell proliferation	*TGFBR1, TGBR3, MMP2, MMP9, IL6, IL6R, TNF, TRIB1, CDKN1A, AIF1, ELN*	2.1E-06
	GO: 0048659	Smooth muscle cell proliferation	*MMP2, MMP9, IL6, IL6R, TNF, TRIB1, CDKN1A, AIF1, ELN*	1.6E-05
**Reactive oxygen species (ROS) metabolic process**	GO: 0027593	Reactive oxygen species metabolic process	*AGTR1, F2, CRP, NOS3, JAK2, TGFBR2, TGFB1, CDKN1A, PKD2, TNF*	0.00093

PCSK9, Proprotein Convertase Subtilisin/Kexin type 9; LIPA, Lipase A; PLTP, Phospholipid Transfer Protein; LRPAP1, Low Density Lipoprotein Receptor Related Protein Associated Protein 1; LPA, Lipoprotein A; LDLR, Low Density Lipoprotein Receptor; SPP1, Secreted Phosphoprotein 1; IL6, Interleukin 6; ANXA2, Annexin A2; AGTR1, Angiotensin II Receptor Type 1; CRP, C Reactive Protein; ABCA1, ATP Binding Cassette Subfamily A Member 1; APOE, Apolipoprotein E; MMP2, Matrix Metallopeptidase 2; MMP3, Matrix Metallopeptidase 3; MMP9, Matrix Metallopeptidase 9; MMP13, Matrix Metallopeptidase 13; LRP1, Low Density Lipoprotein Receptor-related Protein 1; FLOT1, Flotilin 1; TGFBR1, Transforming Growth Factor Beta Receptor 1; TGFB1, Transforming Growth Factor Beta 1; TGFBR3, Transforming Growth Factor Beta Receptor 3; TNF, Tumor Necrosis Factor; TNFSR1, Tumor Necrosis Factor Receptor Superfamily Member 1A; CDKN1a, Cyclin Dependent Kinase Inhibitor 1A; AIF1, Allograft Inflammatory Factor 1; ELN, Elastin; TRIB1, Tribble Pseudokinase 1; F2, Thrombin; NOS3, Nitric Oxide Synthase 3; JAK2, Janus Kinase 2; PKD2, Polycystin 2; Transient Receptor Potential Cation Channel.

**P value of the enrichment is measured by a hyper-geometric test based on GeneMania in-platform algorithm.

### Clinical characteristics of AAA patients

Out of the AAA candidate gene association studies in the literature, we found 15 studies with complete clinical data, totaling a sample size of 10.956 (5676 control & 5280 case). The available clinical data were age (mean), men (n), aortic diameter (mm, mean), smoking (current & past), hypertension, diabetes, CAD (coronary artery disease), PAD (Peripheral Artery Disease), and dyslipidemia. Data variabilities between each variables are different depending on the availability. A detailed summary of each study can be found in Supplementary Table 1.

**Figure 6:**
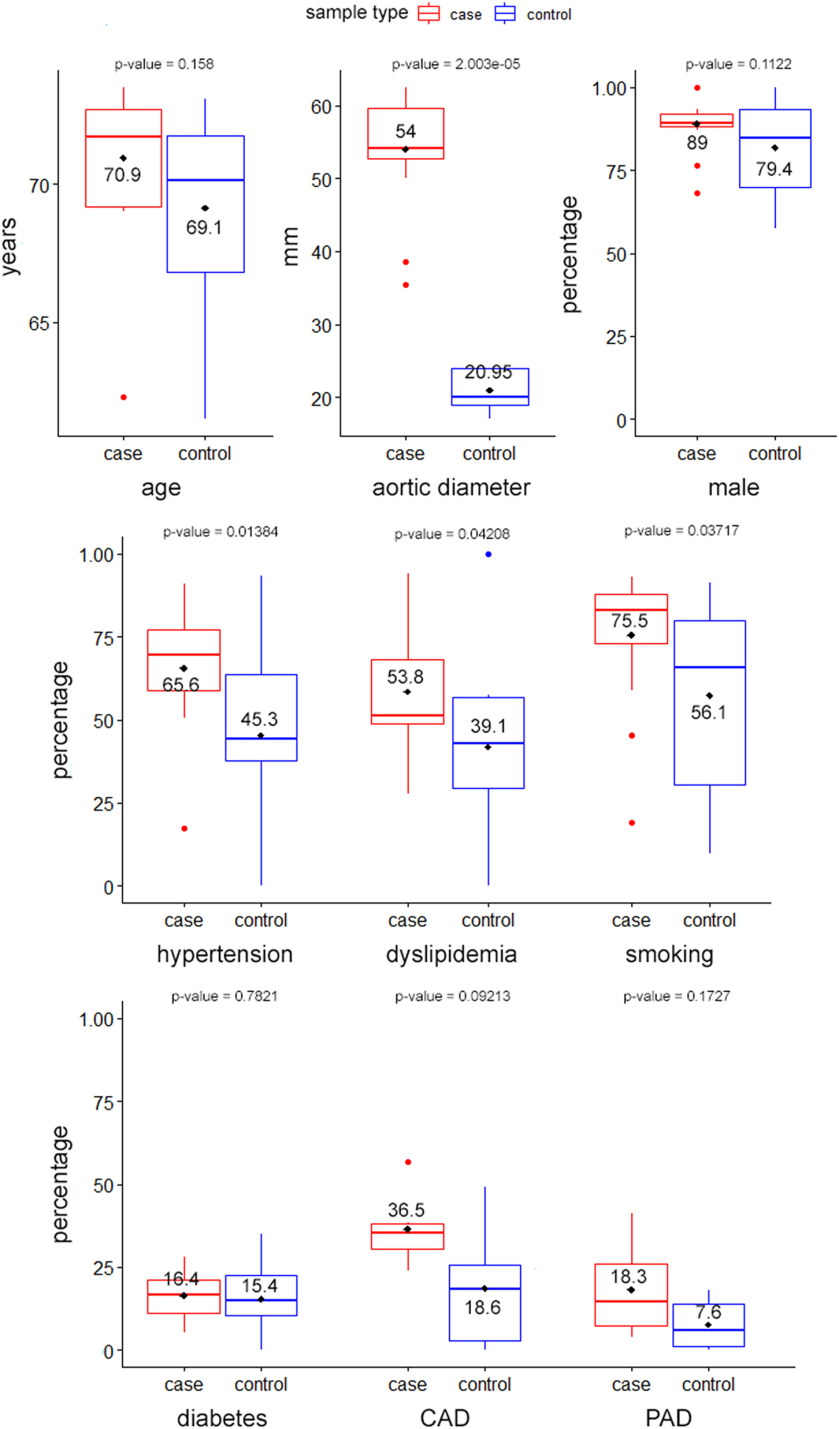
AAA SNPs association with disease phenotype and risk factors. Boxplot representing the association of several identifiable risk factors of AAA between the case group and non-disease controls. Hypertension (*P* = 0.013), dyslipidemia (*P* = 0.042), and smoking (current or history) (*P* = 0.037) are associated with AAA. The sample size consists of 10.956 individuals (5676 non-disease control & 5280 AAA cases).

The non-disease control and AAA case groups shared a similar average age (69.1 and 70.9 years). Several clinical characteristics, such as a history of smoking (past or current, *p* = 0.037), hypertension (*p* = 0.013), and dyslipidemia (*p* = 0.042), were positively associated with AAA. Conversely, there were no significant differences in the presence of diabetes, CAD, and PAD between the two populations. This association is summarized in Fig. 6.

## Discussion

As a complex multifactorial disease, there is little evidence pinpointing the specific genetic predisposition that could contribute to the development of AAA. Previous studies have identified several SNPs associated with AAA, but little is known about the underlying mechanisms and biological significance of the identified SNPs to AAA pathobiology. In this integrative in silico analysis, we used snpXplorer AnnotateMe platform to merge the SNPs candidate from the GWAS catalog and previously published AAA clinical cohorts. We further identify several SNPs with possible pathological signaling pathways associated with AAA as well as their correlative risk factors in these cohorts.

In the present study, we calculated the frequency of the top 20 SNPs in AAA compared to non-disease control and measured the CADD pathogenicity score in order to observe their association with the risk of developing AAA. Here, we identified SNP rs5516, a stop-gained mutation for *KLK1* (Kallikrein 1), with the highest pathogenicity score and a significantly high frequency in AAA (17.8%). Indeed, previous SNPs studies performed in both Australian and Asian cohorts have shown the association of rs5516 with AAA^30,43^. Our analysis identified other interesting polymorphism profiles associated with significant genes that correlated with AAA. We identified a high frequency of rs1800629 for *TNF-α*, and rs1800795 for *IL-6*, inflammatory cytokines well-known to mediate the inflammatory response and smooth muscle cell proliferation during AAA development^44–46^. Indeed, Jablonska et al*.* reported that the mutation detected in both of the alleles increased the risk of AAA formation among heterozygous carriers^31^. However, our result show that CADD score did not necessarily correlate with the frequency of AAA in the observed studies. The SNP rs1801133, located in the coding region of *MTHFR* (Methylenetetrahydrofolate Reductase), was one of the highest-scoring SNPs, yet it’s CT/CC allele was only 3% more expressed in Greek AAA patients than in controls^34^. However, in this study, the analysis of the SNPs in the UK cohort revealed that the frequency of SNP rs1801133 was significantly more frequent. This is most likely due to the population, socio-economical, environmental and genome differences in these two cohorts living in two different geographic areas. Indeed, the meta-analysis performed for SNPs on *MTHFR* showed a discrepancy regarding the protective or pathogenic role of either SNP rs1801133 or *MTHFR*^47–49^. Therefore, this result contextualizes the plasticity of population-specific SNPs and its phenotypic effect to the development of AAA.

Several other SNPs with high frequency in AAA such as rs2230806 (*ABCA1*), rs3775290 (*TLR3*), and rs10757278 (*CDKN2B*) were also strongly associated with significant AAA pathways^50–52^. The loss of *CDKN2B* promotes p53-dependent smooth muscle cell apoptosis and aneurysm formation. In parallel, our analysis revealed high pathogenicity of regulatory *CDKN2B* SNPs (CADD score: 10.42), where this SNP was observed in Chinese and European cohorts^39,53^ and mark the possible genetic-environment interplay in AAA development. However, we were unable to validate its shared associated network in AAA-related pathways along with other gene candidates listed in this study. Therefore, a deeper understanding of the role of *CDKN2B* in AAA is warranted.

The profile of SNPs found in the DNA between genes can act as an essential biological marker with a strong association with the disease. Interestingly, our descriptive analysis conducted from the gene association clinical studies described significant traits related to the well-described risk factors of AAA such as CAD and dyslipidemia pointing a possibility that specific SNPs profile might factor on the progression of these risk factors, and eventually fuel the expansion of AAA. For example, our analysis showed rs429358 polymorphism in *APOE*, a canonical transporter of cholesterol particles^54^, had a significant pathogenicity score in AAA patients. However, this SNP was found to be unassociated with AAA in an Australian cohort^55^. This discrepancy could rely on the characteristics of the cohorts and the type of analysis performed in each study. Genotyping of specific *APOE* alleles was performed in the Australian AAA cohort in 640 samples compared to GWAS population study from 7600 AAA cases using DNA sequencing cross referenced with DNA variants library from European-descent veterans across USA^23^. The sensitivity of the meta-analysis, difference in geographic cohorts studied and the power of the sample size were likely more amenable to capture associations of AAA with rs429358 *APOE* SNPs polymorphism. This further emphasizes the need to perform large-scale worldwide multi-centered studies including populations from disparate groups to uncover the full spectrum of drivers of AAA genetic vulnerability.

Notably, a previous multi-ethnic cohort study has described that the polymorphism of APOE had a significant association with dyslipidemia in Asian ethnic groups^56^. The *APOE* gene is polymorphic and co-exists as APOE-ε2, -ε3 and -ε4 alleles^57^. APOE-ε4 has been shown to associate with increased in LDL-cholesterol levels and higher cardiovascular risk^58^. The highest frequency of APOE-ε4 carrier was observed in the Malay population and was associated with high LDL-C levels. These observations suggest that the inherent geographical genetic background of certain ethnic groups could present a diverse portfolio of SNPs associated with AAA, which warrants further studies.

The main strength of this study is the stringency of our method to include the most recent GWAS catalogs in AAA and combine the data with other available cohorts in the literature to comprehensively identify SNPs with a strong association with AAA. We have summarized and visualized the genomic distribution and frequency of each SNP using the most recent bioinformatic software (snpXplorer)^24^ to identify their associated molecular pathways and variant consequences. We have integrated this result into pathway enrichment analysis to focus on the biological interaction of the associated genes in the AAA pathogenesis.

We acknowledge several limitations in our study. Considering the limited genomic studies performed in AAA, the number of samples included in our analysis is lower than other similar in silico SNPs analysis in other diseases. This limitation hindered us from performing a meta-analysis on each SNPs candidate to validate its consistency between each geographical origin and to confirm the region-specific susceptibility of AAA^59^. Indeed, the small number of cohorts is also a major limitation in performing and assessing the predictive potential of these SNPs candidates in AAA. Moreover, the technological advances in the development of sequencing such as whole-exome sequencing could facilitate the identification of SNPs in the human genomic data. However, this would require significant financial resources and might not be applicable in each clinical setting, thus limiting the potential to use SNPs profile as a predictive marker to identify AAA.

In conclusion, we have identified a significant profile of polymorphism associated with important risk factors such as dyslipidemia and the main pathobiological pathways of AAA development. Further investigation in large population studies will be necessary to confirm this finding and to finally reveal the specific genetic heritage of individuals carrying a risk of developing AAA.

## Supplementary Information

Supplementary Information.

## Data Availability

GWAS datasets analyzed for this study are available in GWAS catalog with the trait label “abdominal aortic aneurysm” (EFO_0004214). The datasets were derived from the following public domain resources: https://www.ebi.ac.uk/gwas/. Non-GWAS studies are openly available at locations cited in the reference section.
