# Supplementary material for: Linking single nucleotide polymorphisms to signaling blueprints in abdominal aortic aneurysms
Source: Sci Rep. 2022 Dec 5;12:20990. doi: 10.1038/s41598-022-25144-y (PMC9722707; doi:10.1038/s41598-022-25144-y)
Supplement: Supplementary file 1 — Supplementary Information. [file 41598_2022_25144_MOESM1_ESM.docx]

**Linking single nucleotide polymorphisms to signaling blueprints in abdominal aortic aneurysms**

Chrysania Lim^1,2*^, Muhammad Yogi Pratama^1,2,3*^, Cristobal Rivera^1,3^, Michele Silvestro^1,3^, Philip S. Tsao^4^, Lars Maegdefessel^5,6,7^, Katherine A. Gallagher^8^, Thomas Maldonado^1^, Bhama Ramkhelawon ^1,3^

^1^Division of Vascular and Endovascular Surgery, Department of Surgery, New York University Langone Medical Center, New York, USA.

^2^Department of Biomedicine, Indonesia International Institute for Life-Sciences (i3L), Jakarta, Indonesia

^3^Department of Cell Biology, New York University Langone Medical Center, New York, USA.

^4^VA Palo Alto Health Care System, Palo Alto, CA & Department of Medicine, Stanford University School of Medicine, Stanford, CA, USA

^5^Department of Vascular and Endovascular Surgery, Technical University Munich, Munich, Germany

^6^German Center for Cardiovascular Research (DZHK), Berlin, Germany, partner site Munich Heart Alliance

^7^Department of Medicine, Karolinska Institute, Stockholm, Sweden

^8^Department of Surgery, University of Michigan, Ann Arbor, MI, USA

- These authors contributed equally to this work

Correspondence should be addressed to BR (bhama.ramkhelawon@nyulangone.org)

**Supplementary Table**

**Supplementary Table 1.** Summary of study participant characteristics from 15 studies

| **Study** | **SNP ID** | **Associated Genes** | **Region** | **Sample size** | | **Age (mean)** | **Men (n)** | **Clinical manifestations (n)** | | | | | | |
| --- | --- | --- | --- | --- | --- | --- | --- | --- | --- | --- | --- | --- | --- | --- |
|  |  |  |  | **Case** | **Control** |  |  | **Aortic dilation (mm)** | **Smoking history** | **Hypertension** | **Dyslipidemia** | **Diabetes** | **Coronary Artery Disease (CAD)** | **Peripheral Artery Disease (PAD)** |
| Galora et al, 2015 | rs1466535, rs6674171, rs3019885 | *LRP1, TDRD10, SLC30A8* | Italy | 423 |  | 73.5 | 376 | 50 | 366 | 302 | 195 | 41 | 163 | 118 |
|  |  |  |  |  |  |  |  |  |  |  |  |  |  |  |
| Saracini et al, 2012 | rs13925, rs1799750, rs2071307, rs2252070, rs2276109, rs2285053, rs243865, rs243866, rs3025058, rs486055, rs4898, rs9619311 | *MMP-9, MMP-1, ELN, MMP-13, MMP-12, MMP-2, MMP-3, MMP-10, TIMP1, TIMP3* |  |  | 423 | 72 | 366 | 19 | 267 | 179 | 50 | 49 | 107 | 67 |
|  |  |  |  |  |  |  |  |  |  |  |  |  |  |  |
| Wei et al, 2014 | rs10757278, rs1333049 | *CDKN2A, CDKN2B* | China | 155 |  | 69.2 | 138 | 54.2 | 132 | 108 | 118 | 18 | 53 | NA |
| Zuo et al, 2015 | rs1800469, rs1626340, rs3773643, rs4522809, rs5182, rs12695895 | *TGFB1, TGFBR1, TGFBR2, AGTR1* |  |  | 310 | 69.5 | 276 | 17 | 167 | 143 | 138 | 46 | 80 | NA |
| Bown et al, 2008 | rs1333049 | *CDKN2A, CDKN2B* | UK | 899 |  | 73 | 814 | 52 | 799 | 528 | 401 | 87 | 262 | NA |
|  |  |  |  |  | 815 | 66 | 798 | <25 | 556 | 309 | 294 | 85 | 94 | NA |
| Baas et al, 2010 | rs10819634, rs1571590, rs1626340, rs764522, rs3087465, rs1036095, rs4522809 | *TGFBR1, TGFBR2* | Netherlands | 736 |  | 71.7 | 663 | 57.3 | 686 | 443 | NA | NA | NA | NA |
|  |  |  |  |  | 1024 | NA | NA | NA | NA | NA | NA | NA | NA | NA |
| Lucarini et al, 2009 |  | *ACE*, *AT1R*, *TGFBR1* | France | 201 |  | 71.5 | 175 | 61 | 140 | 145 | 82 | NA | NA | NA |
|  |  |  |  |  | 252 | 70.6 | 216 | 19 | 45 | 21 | 32 | NA | NA | NA |
| Jabłońska et al, 2020; | rs121917864, rs352139, rs352140, rs3775290, rs3775291, rs3775296, rs5743708, rs5743836 | *TLR2, TLR3, TLR9* | Austria | 104 |  | 70.5 | 93 | 53.6 | 61 | 85 | NA | 21 | 25 | 43 |
| Jabłońska et al, 2021 | rs1800795, rs1800796, rs361525, rs1800629 | *IL-6, TNF-α* |  |  | 112 | 69.7 | 74 | NA | 11 | 0 | NA | 0 | 0 | 0 |
| Zhao et al, 2016 | rs2230806 | *ABCA1* | China | 126 |  | 62.3 | 86 | NA | 24 | 22 | NA | 22 | NA | NA |
|  |  |  |  |  | 119 | 61.5 | 72 | NA | 27 | 46 | NA | 24 | NA | NA |
| Saratzis et al, 2014 | rs3091244 | *CRP* | Greece | 351 |  | 69 | 322 | 62.5 | 257 | 271 | 161 | 77 | NA | 72 |
|  |  |  |  |  | 391 | 73 | 327 | 24 | 311 | 300 | 156 | 88 | NA | 70 |
|  |  |  | UK | 371 |  | 72 | 347 | 54 | 326 | 187 | 226 | 58 | NA | NA |
|  |  |  |  |  | 362 | 71 | 345 | 24 | 302 | 178 | 189 | 52 | NA | NA |
| Crkvenac Gregorek et al, 2016 | rs5186, rs3918242, rs4646994, rs333 | *CCR5, ACE, AT1R, MMP-9* | Croatia | 117 |  | 69 | 102 | NA | 53 | 93 | 65 | 14 | 43 | 8 |
|  |  |  |  |  | 117 | 64 | 67 | NA | 14 | 11 | 2 | 0 | 0 | 0 |
| Rašiová et al, 2021 | rs7635818 | *CNTN3* | Slovak | 166 |  | 72.5 | 147 | 57 | 134 | 151 | 142 | 45 | 94 | NA |
|  |  |  |  |  | 163 | 69.2 | 133 | 18.4 | 149 | 152 | 148 | 44 | 80 | NA |
| Saratzis et al, 2015 | rs3773643, rs3918242, rs2276109, rs1799752, rs1799983, rs1801133 | *MMP-3, MMP-9, MMP-12, ACE, eNOS, MTHFR* | Greece | 397 |  | 69 | 365 | 62 | 290 | 306 | 183 | 87 | NA | 16 |
|  |  |  |  |  | 393 | 72 | 330 | 24 | 314 | 303 | 157 | 90 | NA | 16 |
|  |  |  | UK | 400 |  | 72 | 372 | 54 | 360 | 208 | 252 | 112 | NA | 36 |
|  |  |  |  |  | 400 | 71 | 380 | 24 | 340 | 200 | 212 | 60 | NA | 32 |
| Biros et al, 2011 | rs5516 | *KLK1* | HIMS | 19 |  | 73.2 | 19 | 58.7 | 17 | 12 | 5 | 1 | NA | NA |
|  |  |  |  | 590 |  | 73.3 | 590 | 35.4 | 504 | 315 | 265 | 62 | NA | NA |
|  |  |  |  |  | 695 | 73.1 | 695 | 21.2 | 431 | 263 | 231 | 49 | NA | NA |
|  |  |  | Australia | 60 |  | 72.7 | 53 | 60.5 | 45 | 40 | 29 | 10 | NA | NA |
|  |  |  |  | 165 |  | 71.7 | 126 | 38.5 | 137 | 121 | 114 | 31 | NA | NA |
|  |  |  |  |  | 100 | 65.4 | 64 | 19.9 | 74 | 68 | 53 | 35 | NA | NA |
| Data Availability | | | | | | 100% | 96.875% | 81.25% | 96.875% | 96.875% | 81.25% | 87.5% | 37.5% | 37.5% |


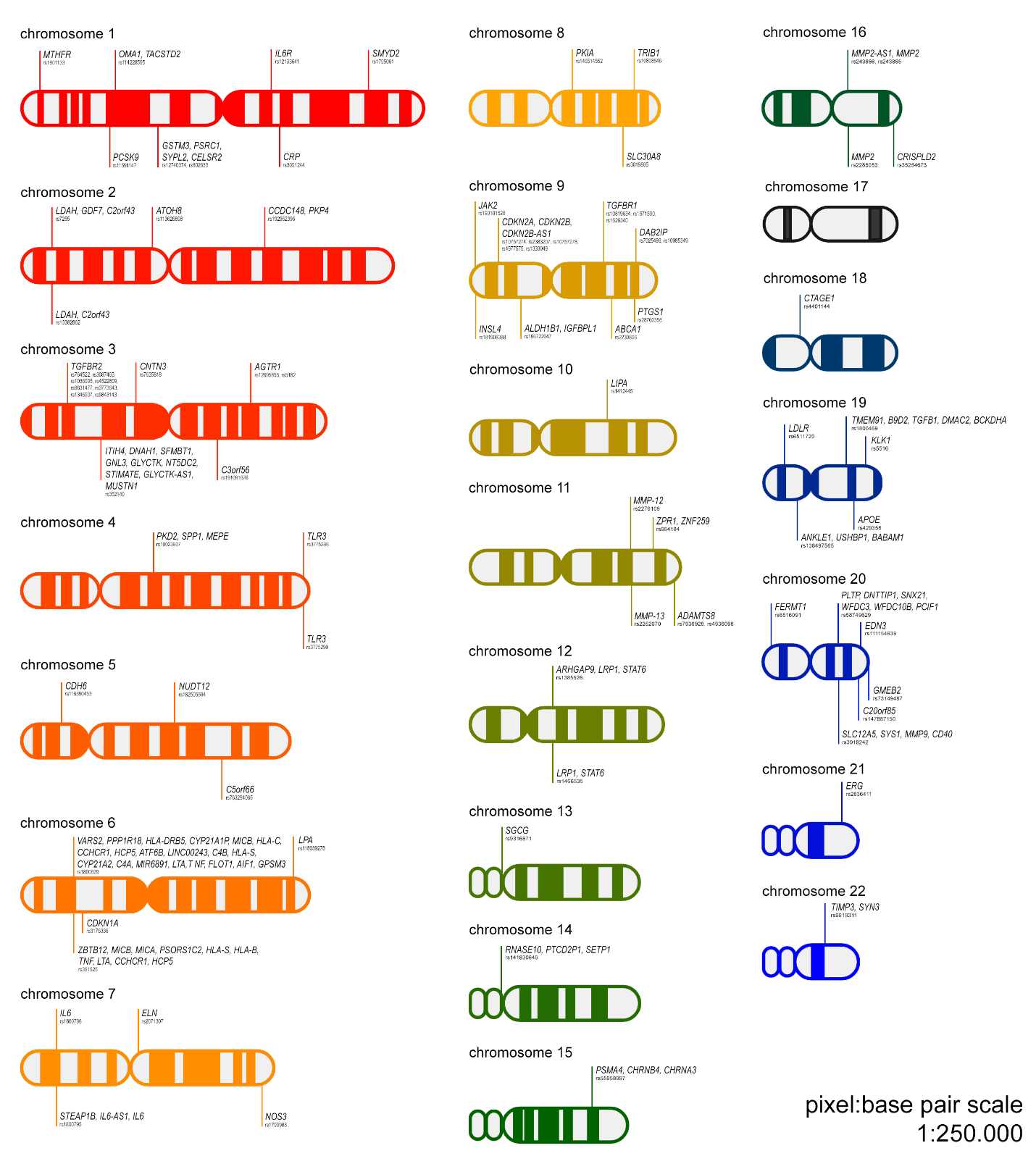


**Supplementary Figure 1.** Full chromosomal mapping of 86 SNPs associated with AAA, in 1:250.000 scale of pixel to base pair.


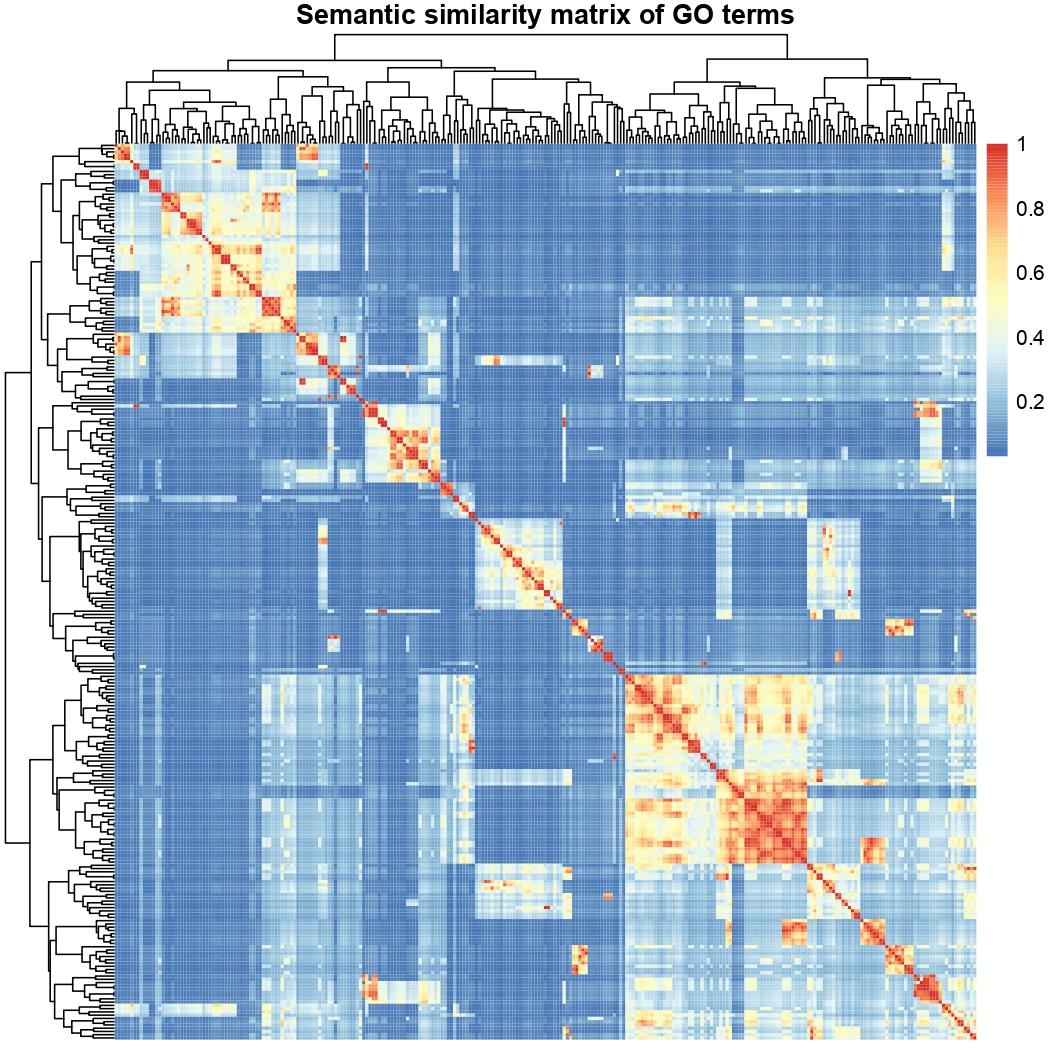


**Supplementary Figure 2.** Semantic-similarity based matrix of gene ontology terms from AAA associated genes. The rows and columns of the matrix represent the different enriched terms. This distance matrix is done using Lin as semantic similarity measure.


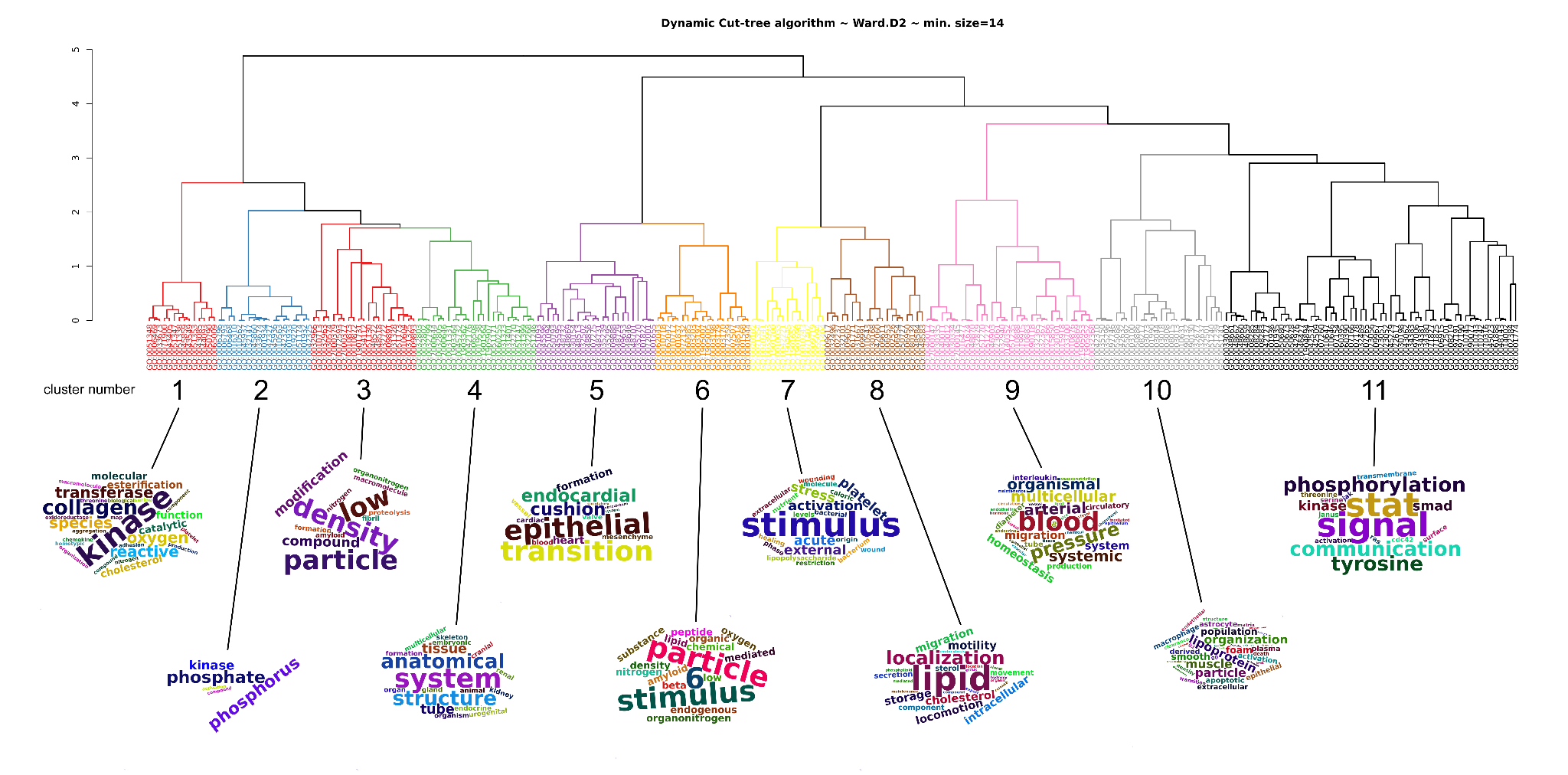


**Supplementary Figure 3.** Dendrogram of gene ontology terms from AAA associated genes semantic-similarity based distance matrix. The number of clusters was calculated using a dynamic cut tree algorithm. The wordcloud visualizes the interpretation of the clusters in the dendrogram. The wordcloud shows only the most frequent terms within each cluster.
